# Prepared health systems provide quality care at all times

**DOI:** 10.1136/bmj-2022-072698

**Published:** 2023-03-13

**Authors:** Blerta Maliqi, Rachael Hinton, Minara Chowdury, Sanam Roder-DeWan, Achama Eluwa, Munir Kassa

**Affiliations:** 1 Policy, Strategy, and Programmes, Department of Maternal, Newborn, Child, Adolescent Health and Ageing, World Health Organization, Geneva, Switzerland; 2 Geneva, Switzerland; 3 Institute for Healthcare Improvement, Dhaka, Bangladesh; 4 Service Delivery Innovation, World Bank, Washington, DC, USA; 5 Community and Family Medicine, Dartmouth Medical School, Hanover, NH, USA; 6 Ministry of Health, Addis Ababa, Ethiopia

## Abstract

**Blerta Maliqi and colleagues** argue that capacity of a health system to provide good quality care even during health crises can save lives and is a strong indication of its resilience

The provision of good quality healthcare is fundamental to population health, preventing avoidable deaths and building population trust in services.^1^ ^2^ ^3^ ^4^ Every year, an estimated eight million people in low and middle income countries die from conditions that would have been treatable with better healthcare provision.^1^ Around half of maternal deaths and 60% of newborn deaths in low and middle income countries could be averted with more effective healthcare.^1^ Improving the ability consistently to provide good quality care—care that is effective, safe, people centred, timely, equitable, integrated, and efficient—is therefore a cornerstone of universal health coverage.

Although most attention has been focused on routine care, provision of good quality care is equally important during health emergencies, such as pandemics, when health systems are stressed. Loss of access to good quality essential services during such events can increase morbidity and mortality from all causes and undermine health systems. For example, evidence suggests that the indirect consequences of health service disruption during the 2014 Ebola outbreak in west Africa—including excess morbidity and mortality from conditions not related to Ebola and diminished public trust in accessing health services—caused more harm than the disease itself.^5^ ^6^ The covid-19 pandemic similarly exposed deficits in the ability of health systems to maintain good quality essential services as attention and resources shifted towards pandemic management and response. 

While the extent to which crises affect health outcomes reflects health systems’ capacity to respond to emergencies, it may also reflect underlying gaps in the provision of good quality essential services.^7^ ^8^ Building the capacity of health systems to learn and adapt will not only help improve services but ensure that they are ready to respond to any challenge and limit the direct and indirect health effects of disruptions.

## Planning for quality matters

The capacity of a health system to continue to provide good quality care even during times of crisis is a strong indication of its resilience.^1^ Various definitions of health system resilience exist, but most agree that resilient health systems, as well as handling health emergencies, are able to maintain good quality essential services while learning and adapting to meet new challenges.^9^ Planning is required to ensure that good quality care is consistently delivered.^10^

Disruptions in the availability and quality of essential health services were ubiquitous during the covid-19 pandemic, especially in the first year, and led to worse health outcomes. Nearly all of the 105 countries surveyed by the World Health Organization between May 2020 and July 2020 reported disruptions in essential health services, with more disruptions in low and middle income countries.^11^ Programmes, including immunisation, maternal and child health, tuberculosis, malaria, and HIV services, recorded gaps in prevention and treatment threatening to reverse gains made towards meeting the Sustainable Development Goals (SDGs).^12^

Although some disruption in the face of crisis is unavoidable, poor outcomes for people with preventive care needs or conditions other than covid-19 resulted from lapses in essential service provision within the overall emergency response. Analysis of routine health information system data from January 2019 to December 2020 in 10 countries, for example, estimates that during the first nine months of the pandemic (March to December 2020), 130 431 fewer women gave birth in a health facility across Ghana, Haiti, Mexico, Nepal, and South Africa, while 131 652 fewer children received their third dose of pentavalent vaccine across Chile, Ethiopia, Mexico, Nepal, and South Africa.^13^ The pandemic response, in combination with pre-existing gaps in health systems’ capacity, meant that there were substantial disruptions in essential health services which resulted in worse health outcomes.^14^ ^15^ ^16^

National responses to the pandemic, including provision of essential health services, were guided by country covid-19 preparedness and response plans. A 2021 analysis of 154 plans from 106 countries found that only 29% explicitly considered the quality of essential health services.^17^ This is an important omission since plans should be in place to ensure that essential health services are maintained to deliver quality care, to save lives, and to preserve community trust even under strict public health prevention measures such as lockdowns or movement restrictions.^8^

The effect of service disruptions on maternal and newborn health outcomes might have been mitigated with better planning of essential services. A prospective observational study among 21 763 women in Nepal conducted over 12.5 weeks before lockdown (1 January to 20 March 2020) and 9.5 weeks during lockdown (21 March to 30 May 2020), for example, found not only that institutional births declined by more than half during the covid-19 lockdown but that the institutional neonatal mortality rate tripled.^18^ This example suggests some of the disruption could have been avoided by better planning.

Pandemic preparedness and planning might also pre-emptively consider the reorganisation of essential services with respect to quality of care standards. During times of crisis, services are often reorganised to manage the emergency. For example, the Nepal study identified reductions in measures of quality such as use of fetal monitoring.^18^ Reorganising services without attention to quality of care may also undermine people’s trust in health services. For example, choice of companionship at birth is an important component of good quality care during childbirth. Despite WHO guidelines recommending that all pregnant women—including those with suspected or confirmed covid-19—should have access to companionship,^19^ many health systems placed restrictions on who could accompany pregnant women during labour and childbirth.^20^ ^21^ This contributed to women’s distrust in health facilities and poor experience of care.^20^ ^22^ A 2020 survey of 1127 maternal and newborn health workers across 71 countries found concerns about quality of care, such as less family involvement and reduced emotional support for women, were similar in many places.^20^

## Learning cultures facilitate adaptation and response

In 2018 the Lancet Global Health Commission on High Quality Health Systems concluded that improving the quality of care will require four system-wide actions to strengthen the foundations of the health system. One of these actions is governance for quality, no matter the context, capacities, or level of health sector development. Governance for quality emphasises the need for health system leadership and management of care quality and calls for developing a health system that is able to learn.^1^ A learning health system helps to strengthen the foundations to deliver high quality care for people when they need it and feeds critical data about quality and outcomes back into efforts to improve its foundations. This learning function becomes particularly important in times of crisis when peoples’ needs, disease burden, and resources change drastically.

Health organisations that had the capacity for learning applied the skills to adapt and reorganise quickly to deliver quality care during the covid-19 pandemic.^23^ ^24^ For example, in May 2020 the #StaffCare Colab Initiative of the provincial department of health in South Africa’s Western Cape province used a series of virtual meetings rapidly to share challenges, learn, and propose solutions to support the health workforce and improve the system across facilities and settings. This built on years of investment that, among other aspects of health systems strengthening, supported the development of a learning culture.^25^

Healthcare teams that were accustomed to using quality improvement methods that are based on continuous adaptation and learning applied them to the emerging problems. In Bangladesh, from April 2020 to August 2020, teams working in 28 learning facilities supported by Unicef across seven districts used their quality improvement skills to manage covid-19 related disruptions to care during childbirth. The disruptions contributed to a 46% increase in perinatal mortality to 84 per 1000 live births. The teams identified, prioritised, and tackled quality issues related to infection prevention and control, triage, and maternal and newborn case management. As a result, by December 2020, mortality reportedly fell to 47 per 1000 live births.^26^

Another example comes from a tertiary care hospital in India, where bed shortages and delays in receiving covid-19 test results resulted in mothers suspected to have covid-19 often being discharged separately from their babies, 2-3 days apart,depriving babies of their mothers’ milk and bonding. Between 1 October 2020 to 25 December 2020, an initiative to change rooming-in practices facilitated maternal-neonatal bonding between mothers with suspected or confirmed covid-19 and their babies, directly improving the quality of care for mothers and neonates.^27^ If all health systems had quality improvement methods routinely embedded many of the covid-19 disruptions could have been ameliorated.

## Creating better prepared systems

Governments, policy makers, practitioners, communities, academics, and partners need to prioritise, invest in, and facilitate development of health systems that deliver good quality care under all circumstances. During health emergencies, good quality routine services can safeguard against direct and indirect deaths not related to the emergency. To achieve the health related targets set out in the SDGs, many governments in low and middle income countries are shifting the focus of their health systems from solely increasing access and coverage of services to achieving universal health coverage of quality care.^2^ In addition to strengthening the foundations of the health system,^1^ this is resulting in the development of national policy and strategy on quality,^10^ along with plans for strengthening capacities and structures within health systems for delivering quality.^3^

 Ethiopia’s approach to the development and implementation of its national quality policy is an example of how to govern, plan, and implement quality of care across all levels and programmes of the health system despite resource constraints.^28^ In response to the covid-19 pandemic and lessons learnt during this period, Ethiopia updated its national strategy in 2022 to include how health services should address quality during crises.^10^ ^29^ Other countries need to develop similar national quality strategies and implementation structures, including response to crises. Governments must also review and update their emergency preparedness and response plans to make provisions for maintaining the quality of service delivery.

The covid-19 pandemic created unprecedented opportunities for reorganising services and introducing innovations. Governments need to capitalise on these opportunities to build systems driven by quality. Quality standards and experience of care should be among the key criteria for reorganising, planning, and implementing essential health services at any time. New initiatives such as that in Kakamega county, Kenya, which aims to improve maternal and newborn survival by redesigning childbirth services so that all births take place in or close to designated hospitals prepared to deliver quality care,^30^ have to be closely documented and the experience shared.

Digital innovations were widely used as part of service reorganisation during the pandemic, but these must also be driven by health outcomes, including patient experience. Recent literature raises concerns that digitalised care may reduce equity, suggesting that the most disadvantaged population groups, such as women of low socioeconomic status, may be at greater risk of exclusion.^16^ ^31^ Evidence is growing on strategies to overcome digital exclusion,^32^ ^33^ but more documentation and research are needed.

The ability to learn is fundamental to delivering quality care and an essential characteristic of a resilient health system.^17^ Systems that have the capacity to learn are able to adapt rapidly. Country reports show that teams used quality improvement methods to adapt to the pandemic context and maintain quality of services. Health system leaders and practitioners have to capitalise on these examples and encourage the continuation of the development of quality improvement capacities and learning across all levels of the health system.^24^ The 2022 WHO health system resilience toolkit clearly identifies quality of care and quality improvement as central to maintaining coverage of good quality routine health services during public health emergencies.^34^ Stakeholders involved in strengthening health systems must consult and prioritise these resilience building actions to ensure that quality services are maintained in all settings at all times. However, it remains to be seen if countries will use this guidance to inform the planning and development of their health systems.

Quality of care saves lives and strengthens the resilience of a health system. Planning for and enabling health systems to consistently deliver quality care even during a crisis or through an abrupt reorganisation should not be an afterthought. Healthcare emergencies will continue to occur beyond the covid-19 pandemic. Efforts and investments to ensure that quality is well embedded in the national health strategies and operations, including the emergency preparedness and response plans, must continue.

**Key messages**

- Quality of care was an afterthought in both pre-pandemic planning and reorganisation of essential services during the covid-19 crisis
- Health system leaders must ensure that national strategies on quality of care are aligned and integrated with emergency preparedness and response plans so that quality is a constant priority
- Health organisations and teams that are able to constantly learn and adapt are able to deliver quality care and are more resilient

## Notes

Contributors and sources: All authors have experience working in maternal, newborn, child, and adolescent health. BM and RH developed the initial outline of the paper and reviewed the relevant literature. BM led the writing of the paper, and together with RH wrote the first draft. SRD, MC, MK, and AE reviewed and provided written inputs on the first draft as well as additional literature. BM finalised the draft with RH and all co-authors reviewed and agreed with the final version. BM is the guarantor. The authors alone are responsible for the views expressed in this article and they do not necessarily represent the views, decisions, or policies of the institutions with which they are affiliated.

Provenance and peer review: Commissioned; externally peer reviewed.

This article is part of a collection proposed by the World Health Organization and the World Bank and commissioned by *The BMJ*. *The BMJ* peer reviewed, edited, and made the decision to publish these articles. Article handling fees are funded by the Bill & Melinda Gates Foundation. Jennifer Rasanathan, Juan Franco, and Emma Veitch edited this collection for *The BMJ*. Regina Kamoga was the patient editor.
